# The Change of Noncoding RNA Expression in Olfactory Bulb of Hepatic Encephalopathy Mouse Model: Transcriptomic Analysis and Cellular Analysis

**DOI:** 10.1111/cns.70596

**Published:** 2025-09-04

**Authors:** Young‐Kook Kim, Sujung Yeom, Seo Yoon Choi, Yeongseo Ryu, Dahee Jeong, Danbi Jo, Dong Hoon Lee, Juhyun Song

**Affiliations:** ^1^ Biomedical Science Graduate Program (BMSGP) Chonnam National University Hwasun Republic of Korea; ^2^ Department of Biochemistry Chonnam National University Medical School Hwasun Republic of Korea; ^3^ Department of Otolaryngology‐Head and Neck Surgery Chonnam National University Medical School & Hwasun Hospital Hwasun Republic of Korea; ^4^ Department of Anatomy Chonnam National University Medical School Hwasun Republic of Korea

**Keywords:** bile duct ligation, hepatic encephalopathy, noncoding RNAs, olfactory bulb, RNA sequencing

## Abstract

**Objectives:**

Hepatic encephalopathy (HE) is a neuropsychiatric disorder associated with cirrhosis and chronic liver disease primarily driven by ammonia (NH3) toxicity, which leads to neuroinflammation and cognitive deficits. Recent studies have identified olfactory dysfunction as a potential early indicator of HE, linked to ammonia‐induced neurotoxicity in the brain.

**Methods:**

After confirming physiological alterations in olfactory cells induced by ammonia, we assessed gene expression changes in olfactory bulbs of bile duct ligation (BDL) mice as an HE mouse model. We systematically profiled diverse coding and noncoding RNAs (ncRNAs) associated with olfactory dysfunction in HE and analyzed the functional implications based on transcriptomic signatures. We also compared ammonia toxicity effects between the olfactory bulb and cerebral cortex in this animal model.

**Results:**

Furthermore, we investigated the differential impacts on the olfactory bulb between HE and high‐fat diet‐induced models, two major paradigms of metabolic imbalance. We identified key RNAs commonly altered between the olfactory bulb and cerebral cortex of the HE model, as well as in olfactory bulbs across BDL and high‐fat diet models.

**Conclusions:**

Our results provide a transcriptomic resource for understanding the molecular landscape of HE‐related olfactory dysfunction and may inform future studies aimed at functional validation and therapeutic exploration.

## Graphical abstract

Bile duct ligation (BDL) surgery induced hepatic encephalopathy mouse model. We took out olfactory bulb in BDL mouse, and subsequently performed RNA sequencing using olfactory bulb. We found significantly changed protein‐coding RNA, long noncoding RNAs (lncRNAs), and circular RNAs (circRNAs). We cultured OBC1 olfactory bulb cells under hyperammonia in vitro condition. We observed increased level of ROS, increased production of pro‐inflammatory cytokines, and increased mitochondrial dysfunction, and increased cell death signal in OBC1 cells under hyperammonia condition.

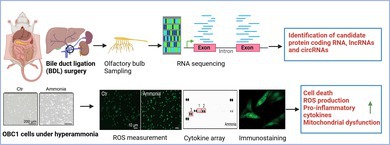

**Abbreviations**
*Bach2*
: BTB domain and CNC homolog 2
BDL: bile duct ligation
*Calb1*
: calbindin 1
CC3: Cleaved Caspase‐3
*Chrna4*
: cholinergic receptor nicotinic alpha 4 subunit
*Chrna4*
: Cholinergic Receptor Nicotinic Alpha 4 Subunit
circRNAs: circular RNAs
*Cnr1*
: cannabinoid receptor 1
CSF: cerebrospinal fluid
DAPI: 4′,6‐diamidino‐2‐phenylindole
DCF‐DA: 2′,7′‐dichlorofluorescein diacetate
FPKM: fragments per kilobase of transcript per million mapped reads
*GAS5*
: *Growth Arrest Specific 5*
GO: Gene Ontology
*HAUS3*
: HAUS augmin‐like complex subunit 3
HE: Hepatic encephalopathy
HFD: high‐fat diet
*Icam1*
: intercellular adhesion molecule 1
*Jak3*
: Janus kinase 3
JC‐1: 5,5′,6,6′‐tetrachloro‐1,1′,3,3′‐tetraethylbenzimidazolocarbo‐cyanine iodide
*Kcnk4*
: potassium two pore domain channel subfamily K member 4
lncRNAs: long noncoding RNAs
*Lrg1*
: leucine‐rich alpha‐2 glycoprotein 1
MCP1: monocyte chemoattractant protein 1
M‐CSF: macrophage colony‐stimulating factor
*Mgst1*
: microsomal glutathione S‐transferase 1
MHE: minimal hepatic encephalopathy
MMP: mitochondrial membrane potential
MRP‐14: myeloid‐related protein 14
MSigDB: Molecular Signatures Database
ncRNAs: Noncoding RNAs
*Neurod2*
: neuronal differentiation 2
NH3: ammonia
*Nudt5*
: Nudix Hydrolase 5
RAGE: receptors for advanced glycation end‐products
*Rbm3*
: RNA binding motif protein 3
*Rnft1*
: Ring finger protein transmembrane 1
ROS: Reactive oxygen species
*S100A8*
: S100 calcium binding protein A8
*S100a9*
: S100 calcium‐binding protein A9
*Slc27a3*
: solute carrier family 27 member 3
*Socs3*
: suppressor of cytokine signaling 3
*Tagln*
: Transgelin
TARC: thymus and activation‐regulated chemokine
TLR4: toll‐like receptor 4
*Ttr*
: transthyretin
*ZFAS1*
: ZNFX1 Antisense RNA 1

## 1 Introduction

Hepatic encephalopathy (HE) is a neuropsychiatric disorder resulting from liver dysfunction with/without portal shunts, commonly associated with cirrhosis and chronic liver disease [1]. Patients with HE exhibit a spectrum of cognitive deficits, along with motor and sensory impairments [2, 3]. Although HE involves multiple factors disrupting neuronal function, ammonia (NH3) stands as the central pathophysiological driver. This nitrogenous toxin, produced in the intestine through bacterial breakdown of urea, becomes problematic in cirrhotic patients due to compromised liver function and shunting of ammonia‐laden blood into systemic circulation. Upon crossing the blood–brain barrier, ammonia is converted to glutamine within astrocytes, triggering cellular swelling, oxidative stress, and neurological dysfunction [1, 4, 5, 6]. Emerging evidence indicates that olfactory dysfunction may be significantly affected by liver disease. Temmel et al. reported olfactory impairment in patients with liver cirrhosis, suggesting that olfactory deficits could serve as an early indicator of HE [7]. Similarly, Zucco et al. found that patients with minimal hepatic encephalopathy (MHE), the earliest stage of HE, demonstrated significant olfactory deficits, indicating a relationship between MHE and alterations in HE‐related neurotransmitter function [8]. Heiser et al. further established that olfactory function is notably compromised in cirrhotic patients, with dysfunction severity correlating with HE progression [9]. Recent animal studies revealed that acute hyperammonemia increases mitral cell excitability in the olfactory bulb by enhancing glutamate receptor activity and recruitment to the cell membrane. This heightened excitability subsequently leads to neuronal excitotoxicity and cell death, suggesting that ammonia‐induced overactivation of glutamate receptors contributes to the olfactory deficits observed in conditions like HE [10].

The olfactory system originates in the olfactory mucosa, where odor information is transmitted to the olfactory bulb. This six‐layered structure receives axons from olfactory receptor neurons that form the olfactory nerve layer. Mitral and tufted cells connect to olfactory bulb glomeruli, while granule cells function as interneurons. The axons of mitral and tufted cells constitute the lateral olfactory tract, extending to various brain regions collectively known as the primary olfactory cortex, including the anterior olfactory nucleus, piriform cortex, olfactory tubercle, lateral entorhinal cortex, and paraamygdaloid complex. Neurons in the primary olfactory cortex project to secondary olfactory targets, such as the hypothalamus, thalamus, orbitofrontal cortex, insular cortex, and dorsal hippocampus [11, 12, 13, 14].

The human olfactory bulb demonstrates remarkable plasticity and adaptability, though the precise mechanisms underlying this plasticity remain incompletely understood. The olfactory bulb may undergo changes in size and function in response to sensory inputs and during recovery from disorders, potentially through ongoing neurogenesis. This plasticity appears influenced by both “centripetal” and “centrifugal” inputs, enabling the olfactory bulb to actively process and refine olfactory information. Magnetic resonance imaging (MRI) studies have demonstrated that olfactory bulb volume correlates with olfactory function [15, 16, 17]. Toxic conditions such as HE can damage the olfactory epithelium and injure the main olfactory bulb, resulting in decreased synaptic plasticity. Since the olfactory bulb innervates the primary olfactory cortex, which encompasses various brain regions, compromised olfactory bulb function may adversely affect cognitive processes and memory [18, 19]. Previous studies have shown that bile duct ligation (BDL) in mice induces hyperammonemia, leading to glial cell activation—particularly in astrocytes and microglia—and neuroinflammation. This results in motor dysfunction, increased blood–brain barrier permeability, and metabolic disturbances in the brain, including elevated glutamine and bile acid accumulation [20]. Additionally, HE impairs glymphatic clearance in the brain, including the olfactory bulb, due to reduced aquaporin‐4 expression in rat models. This dysfunction may contribute to cognitive and olfactory deficits in HE [21].

Our previous research investigated the role of p53 in HE using human neuroblastoma cells treated with ammonium chloride to mimic hyperammonemia, focusing on two long non‐coding RNAs (lncRNAs), ZFAS1 and GAS5, and their knockdown effects [19]. Noncoding RNAs (ncRNAs), produced from regions outside protein‐coding genes in the genome, regulate gene expression and protein activity. As ncRNAs have been identified as crucial regulators in various diseases, their modulation represents a promising therapeutic approach [22, 23, 24].

In this study, we aimed to identify ncRNAs associated with olfactory dysfunction induced by HE. We analyzed gene expression changes in the olfactory bulb of the BDL mouse model of HE and examined cellular alterations in OBC1 olfactory bulb cells under hyperammonia conditions. Our findings provide a transcriptomic landscape of HE‐associated olfactory dysfunction and may inform future studies aiming to explore the functional roles of specific ncRNAs and their therapeutic potential.

## 2 Materials and Methods

### 2.1 Bile Duct Ligation (BDL) Mouse Model

Male C57BL/6J mice (12 weeks old; Koatech, Seoul, Korea) were housed in the Laboratory Animal Research Center at Chonnam National University (CNU). Animals were maintained under controlled environmental conditions (23°C, 60% ± 10% relative humidity) with a 16/8 h light/dark cycle and provided ad libitum access to standard laboratory chow and water throughout the experimental period. To establish the HE model, mice underwent either a sham operation or BDL operation. Anesthesia was induced via intraperitoneal injection of 2,2,2‐tribromoethanol/2‐methyl‐2‐butanol (0.2 mg/g body weight; Sigma‐Aldrich, MO, USA) and maintained with 1.5%–2.0% isoflurane in an air/oxygen mixture. BDL was performed using a 5‐0 silk suture. The experimental endpoint was set at 2 weeks post‐surgery. All experimental procedures adhered to the “96 Guidance for Animal Experiments” and were approved by the CNU Institutional Animal Care and Use Committee (approval number: CNU IACUC‐H‐2022‐8).

### 2.2 Cell Culture

OBC1 olfactory bulb cells (ABM, Toronto, ON, Canada) [25] were cultured at a density of 2 × 10^4^ cells/cm^2^ in PriGrow III Medium (TM003; ABM, Toronto, ON, Canada) supplemented with 10% fetal bovine serum (FBS; Millipore, MO, USA) and 100 U/mL penicillin–streptomycin (Thermo Fisher Scientific, St. Louis, MA, USA). Culture medium was replaced every 48 h. For ammonia treatment conditions, cells were exposed to 20 mM ammonia for 24 h to simulate hyperammonemia.

### 2.3 Measurement of Mitochondrial Membrane Potential (MMP)

Mitochondrial membrane potential (MMP ΔΨm) was performed by using fluoroprobe 5,5′,6,6′‐tetrachloro‐1,1′,3,3′‐tetraethylbenzimidazolocarbo‐cyanine iodide (JC‐1) according to the manufacturer's instructions (Abcam, ab113850, Waltham, MA, USA). Briefly, 8 × 10^3^ OBC1 cells were seeded in 96‐well plates and then treated with ammonia (20 mM) for 24 h. The next day, the cells were washed with 1× PBS, 50 μL of the medium containing 5 μM JC‐1 was added to the wells, and then incubated at 37°C for 30 min in the dark. After the reaction was completed, the medium with JC‐1 was gently removed. The cells were washed twice with 1× dilution buffer included in the kit, and 100 μL of 1× dilution buffer was added to each well. Images of the stained cells were captured using the Eclipse Ts2 fluorescence microscope (Nikon, Tokyo, Japan) with a 200× objective, and fluorescent images were analyzed by utilizing Image J software (V.1.54 g). Mitochondrial membrane potential was indicated by the green‐to‐red fluorescence intensity ratio.

### 2.4 Reactive Oxygen Species (ROS) Measurement

Intracellular reactive oxygen species (ROS) levels were quantified using 2′,7′‐dichlorofluorescein diacetate (DCF‐DA, Sigma‐Aldrich, MO, USA). OBC1 cells (1.4 × 10^5^) were seeded in 6‐well plates and exposed to 20 mM ammonia for 24 h. Following treatment, the culture medium was replaced with medium containing 10 μM DCF‐DA, and cells were incubated at 37°C for 20 min. After incubation, cells were thoroughly washed with PBS, and 100 μL of PriGrow III medium was added to each well. Fluorescence images were acquired using an Eclipse Ts2 Fluorescence microscope (Nikon, Tokyo, Japan). For quantitative analysis, fluorescence intensity was measured at excitation wavelength 485 nm and emission wavelength 530 nm using a SpectraMax M2 microplate reader (Molecular Devices, CA, USA).

### 2.5 Cytokine Array

Plasma cytokine profiling was conducted using the Mouse Cytokine Array Kit (ARY006; R&D Systems, MN, USA). Culture media from control and ammonia‐treated cells were collected for plasma isolation. The cytokine detection membrane was first blocked with Array Buffer 6 for 1 h at room temperature. Media samples were diluted in Array Buffer 4 and incubated with the membrane overnight at 4°C with gentle agitation. Following this incubation, the membrane was treated for 1 h at room temperature with a detection antibody cocktail prepared by mixing Array Buffer 4 and Array Buffer 6 according to the manufacturer's protocol. The biotinylated detection antibody mixture was reconstituted with distilled water immediately before use to ensure optimal activity.

After antibody incubation, the membrane was treated with streptavidin‐horseradish peroxidase (HRP) for 30 min at room temperature. Signal detection was performed using chemiluminescent reagents, and images were captured using Fusion Solo software (version 16.0.8.0; Vilber, Collégien, France). Comprehensive densitometric analysis was performed using ImageJ software (version 1.54d; National Institutes of Health, Bethesda, MD, USA), with the pixel density of each spot normalized to reference spots on the array to enable accurate quantitative comparison between experimental conditions.

### 2.6 RNA Sequencing and Functional Analysis of Changed Genes

Total RNA was extracted from the olfactory bulbs of sham‐operated and BDL mice using TRIzol reagent (Takara, Nojihigashi, Japan) according to the manufacturer's protocol. Genomic DNA contamination was eliminated through DNase I treatment (Takara, Nojihigashi, Japan). RNA integrity and quality were assessed using an Agilent 2100 Bioanalyzer (Agilent Technologies, Santa Clara, CA, USA). RNA‐sequencing libraries were prepared using the TruSeq Stranded Total RNA Kit (Illumina, San Diego, CA, USA) and sequenced on a NovaSeq 6000 System (Illumina, San Diego, CA, USA). Raw sequence reads underwent quality filtering and trimming using Trimmomatic [26]. The processed reads were aligned to the mouse reference genome (mm10) using STAR aligner [27]. Gene expression levels were quantified as fragments per kilobase of transcript per million mapped reads (FPKM) using Cuffnorm, based on GENCODE annotation (Release M23, GRCm38.p6) [28]. For subsequent analyses, we excluded genes with average FPKM values less than 1 across all samples or equal to 0 in any sample to ensure reliability of expression data. Differential gene expression analysis between sham and BDL groups was performed using Student's *t*‐test, with the statistical significance threshold set at *p* < 0.01. This criterion identified 276 differentially expressed genes, which were then subjected to Gene Ontology (GO) enrichment analysis using the Molecular Signatures Database (MSigDB v7.5.1) [29] to identify functional pathways affected by BDL operation. Pathway enrichment analysis was performed using the WikiPathways 2024 Mouse gene set library via the Enrichr web tool (https://maayanlab.cloud/Enrichr) [30, 31]. Increased genes from both the olfactory bulb and cerebral cortex were analyzed, and significantly enriched pathways were identified based on adjusted *p* values (Benjamini‐Hochberg adjusted *p* values < 0.01). For the genes with the most significantly altered expression in the olfactory bulb after BDL operation, we constructed a protein interaction network focusing on the top 30 genes ranked by *p* values. The network analysis was conducted using GeneMANIA plugins [32] (http://www.genemania.org/plugin/) implemented in Cytoscape [33] (version 3.9.1), allowing visualization of functional relationships among the differentially expressed genes.

### 2.7 Western Blot Analysis

OBC1 cells were lysed using ice‐cold RIPA buffer (Biosesang, Seoul, Korea) supplemented with protease and phosphatase inhibitors (GenDEPOT). The lysates were incubated on ice for 30 min and centrifuged at 14,000 × g for 15 min at 4°C. The supernatants were collected, and protein concentration was determined using the Pierce BCA Protein Assay kit (Thermo Fisher Scientific, St. Louis, MO, USA). For each sample, 20 μg of protein was separated by electrophoresis on a 12% SDS‐polyacrylamide gel. Proteins were subsequently transferred to a polyvinylidene difluoride (PVDF) membrane (Merck Millipore, Burlington, MA, USA) preactivated with methanol (Merck Millipore, Burlington, MA, USA).

Membranes were blocked with 5% BSA (GenDEPOT) for 1 h at room temperature and then incubated overnight at 4°C with specific primary antibodies against LC3B (1:1000, Cell Signaling Technology, #3868, Danvers, MA, USA) and GAPDH (1:4000, Santa Cruz, sc‐32,233, Dallas, TX, USA). Following primary antibody incubation, membranes were washed three times with 1× TBS‐T (Tris‐buffered saline containing 0.1% Tween 20) and incubated with horseradish peroxidase‐conjugated secondary antibody (1:5000) for 1 h at room temperature. Protein bands were visualized using Immobilon Western Chemiluminescent HRP Substrate solution (Millipore, St. Louis, MO, USA) and detected with Fusion Solo imaging system (Vilber, Lemont, IL, USA). Quantitative analysis of band intensity was performed using ImageJ software (v1.54d), with LC3B band intensity normalized to GAPDH as a loading control.

### 2.8 Immunocytochemistry

OBC1 cells (2 × 10^4^ cells/cm^2^) were seeded onto coated coverslips in culture plates. Cells were fixed with 2% paraformaldehyde (PFA) for 10 min at room temperature, followed by three washes with 1× PBS. For immunostaining, cells were incubated overnight at 4°C with primary antibodies diluted 1:200 in PBS containing 0.3% Triton X‐100 to facilitate membrane permeabilization. The primary antibodies used in this study included Bax (Cell Signaling Technology, #2772, Danvers, MA, USA) and Cleaved Caspase‐3 (CC3) (Cell Signaling Technology, #9661, Danvers, MA, USA). After overnight incubation, cells were washed three times with 1× PBS and subsequently incubated with Alexa Fluor 488‐conjugated anti‐rabbit IgG secondary antibodies (Santa Cruz Biotechnology) diluted 1:200 in PBS containing 0.3% Triton X‐100 for 1 h at room temperature. Cell nuclei were counterstained with mounting medium containing 4′,6‐diamidino‐2‐phenylindole (DAPI). Immunolabeled cells were visualized using a Carl Zeiss LSM 900 with Airyscan2 confocal microscope (Carl Zeiss, White Plains, NY, USA). Fluorescence intensity was quantified using ImageJ software (version 1.54d; National Institutes of Health, Bethesda, MD, USA).

### 2.9 Statistical Analysis

For experiments with sample size *n* ≥ 4 (cell‐based assays), we assessed data normality using the Shapiro–Wilk test. If the data significantly deviated from a normal distribution (*p* < 0.05), we used the Mann–Whitney *U* test. Otherwise, we performed the Student's *t*‐test. For RNA‐seq data (*n* = 3 per group), normality testing was not feasible due to the small sample size. Therefore, we log_2_‐transformed FPKM values and assumed approximate normality, following standard practice in transcriptomic studies, and used the Student's *t*‐test to assess differential gene expression. The data are presented as group means ± standard error of the mean (SEM). Statistical analysis was performed using GraphPad Prism 8 (GraphPad Software Inc., San Diego, CA, USA). Statistical significance was established at *p* < 0.05 unless indicated otherwise.

## 3 Results

### 3.1 Ammonia Toxicity Aggravates Mitochondrial Dysfunction, Cell Death, ROS Generation, and the Secretion of Pro‐Inflammatory Cytokines in OBC1 Olfactory Bulb Cells

**FIGURE 1:**
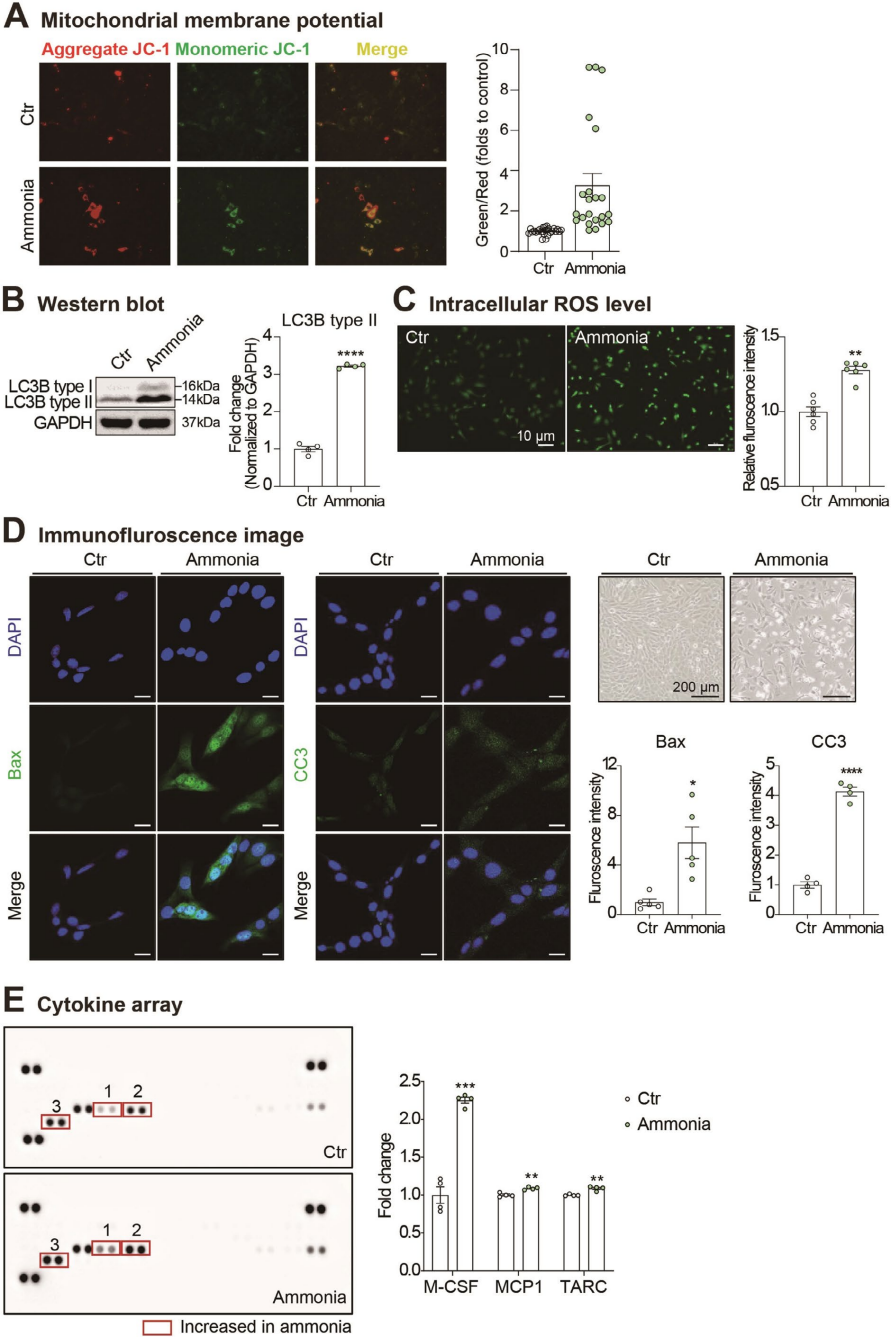
Effects of hyperammonemia on mitochondrial function, cell death, ROS generation, and cytokine release in OBC1 olfactory bulb cells. (A) Measurement of mitochondrial membrane potential (MMP ΔΨm) with the ratio of JC‐1 monomers (green) to JC‐1 aggregates (red) in OBC1 cells treated with ammonia (Control, *n* = 26; Ammonia treat, *n* = 22). Control indicates no treatment, and ammonia indicates ammonia (20 mM) treatment. Following staining with JC‐1 and acquiring equivalent red and green images, ratios of JC‐1 monomers (green) to JC‐1 aggregates (red) fluorescence intensities were calculated and displayed in the graphs as foldchange (FC) of the treatment versus the control. Data are displayed as mean ± SEM. (A) Mann–Whitney *U* test was used for statistical analysis (****p < 0.0001). (B) Western blot analysis of LC3B protein expression in OBC1 cells following 24‐h ammonia exposure. LC3B type II levels were normalized to GAPDH as loading control. Statistical significance was determined using Welch's *t*‐test (*n* = 4, ****p < 0.0001). (C) Representative fluorescence images and quantitative analysis of intracellular reactive oxygen species (ROS) accumulation in ammonia‐treated OBC1 cells visualized using 2′,7′‐dichlorofluorescein diacetate (DCF‐DA) fluorescent probe. ROS levels were quantified based on DCF fluorescence intensity. A Mann–Whitney *U* test was used for statistical analysis (*n* = 6, **p < 0.01). (D) Immunofluorescence detection of apoptotic markers Bax and cleaved caspase‐3 (CC3) in control (Ctr) and ammonia‐treated cells (Scale bar: 20 μm). Nuclei are counterstained with 4′,6‐diamidino‐2‐phenylindole (DAPI) (blue). Enhanced expression of Bax and CC3 (green) is evident in ammonia‐treated cells. Corresponding phase‐contrast microscopic images are shown on the right. Fluorescence intensity quantification is presented (Bax: *N* = 4, CC3: *N* = 5). Statistical significance was determined using Welch's *t*‐test (*p < 0.05, ****p < 0.0001). (E) Cytokine array analysis of conditioned media from control and ammonia‐treated cells. Pro‐inflammatory cytokines macrophage colony‐stimulating factor (M‐CSF), monocyte chemoattractant protein 1 (MCP1) and thymus and activation‐regulated chemokine (TARC) showed significant increases in ammonia‐treated cells (highlighted in red boxes). Data are presented as mean ± SEM. Statistical significance was determined using Welch's *t*‐test (**p < 0.01, ***p < 0.001).

To investigate the effects of ammonia toxicity on olfactory bulb cell physiology, we assessed multiple cellular parameters in ammonia‐treated OBC1 cells, an immortalized mouse olfactory bulb cell line (Figure 1). We first evaluated mitochondrial function using a mitochondrial membrane potential assay. Exposure to ammonia significantly compromised mitochondrial function in OBC1 cells, as evidenced by disrupted membrane potential (Figure 1A). We then examined the impact of ammonia treatment on autophagy by measuring LC3B protein levels. Western blot analysis revealed a significant increase in LC3B type II expression in ammonia‐treated OBC1 cells compared to untreated controls, indicating enhanced autophagosome formation (Figure 1B). To confirm the induction of oxidative stress, we assessed intracellular ROS levels using the fluorescent probe DCF‐DA. Fluorescence microscopy revealed markedly increased ROS accumulation in ammonia‐treated OBC1 cells. Quantitative analysis of fluorescence intensity further confirmed that ROS levels were significantly elevated following ammonia exposure (Figure 1C). These findings collectively indicate that ammonia treatment induces substantial oxidative stress, which subsequently triggers autophagy activation in OBC1 cells. As anticipated, ammonia treatment induced significant cell death in olfactory bulb cells, demonstrated by increased expression of apoptotic markers including CC3 and Bax (Figure 1D). To characterize the inflammatory response of olfactory bulb cells to ammonia toxicity, we analyzed the secretion profile of various cytokines using a cytokine array. Our data revealed that ammonia exposure significantly increased the production of pro‐inflammatory cytokines, particularly macrophage colony‐stimulating factor (M‐CSF), monocyte chemoattractant protein 1 (MCP1), and thymus and activation‐regulated chemokine (TARC) from OBC1 olfactory bulb cells (Figure 1E). Collectively, these findings demonstrate that exposure to elevated ammonia levels—a hallmark of HE—leads to multiple cellular pathologies in olfactory bulb cells, including impaired mitochondrial membrane potential, enhanced ROS generation, increased cell death, and amplified secretion of pro‐inflammatory cytokines. These cellular alterations likely contribute to olfactory dysfunction observed in HE patients.

### 3.2 BDL Operation Triggers the Change of Protein‐Coding RNA Expression in the Olfactory Bulb Tissue

**FIGURE 2:**
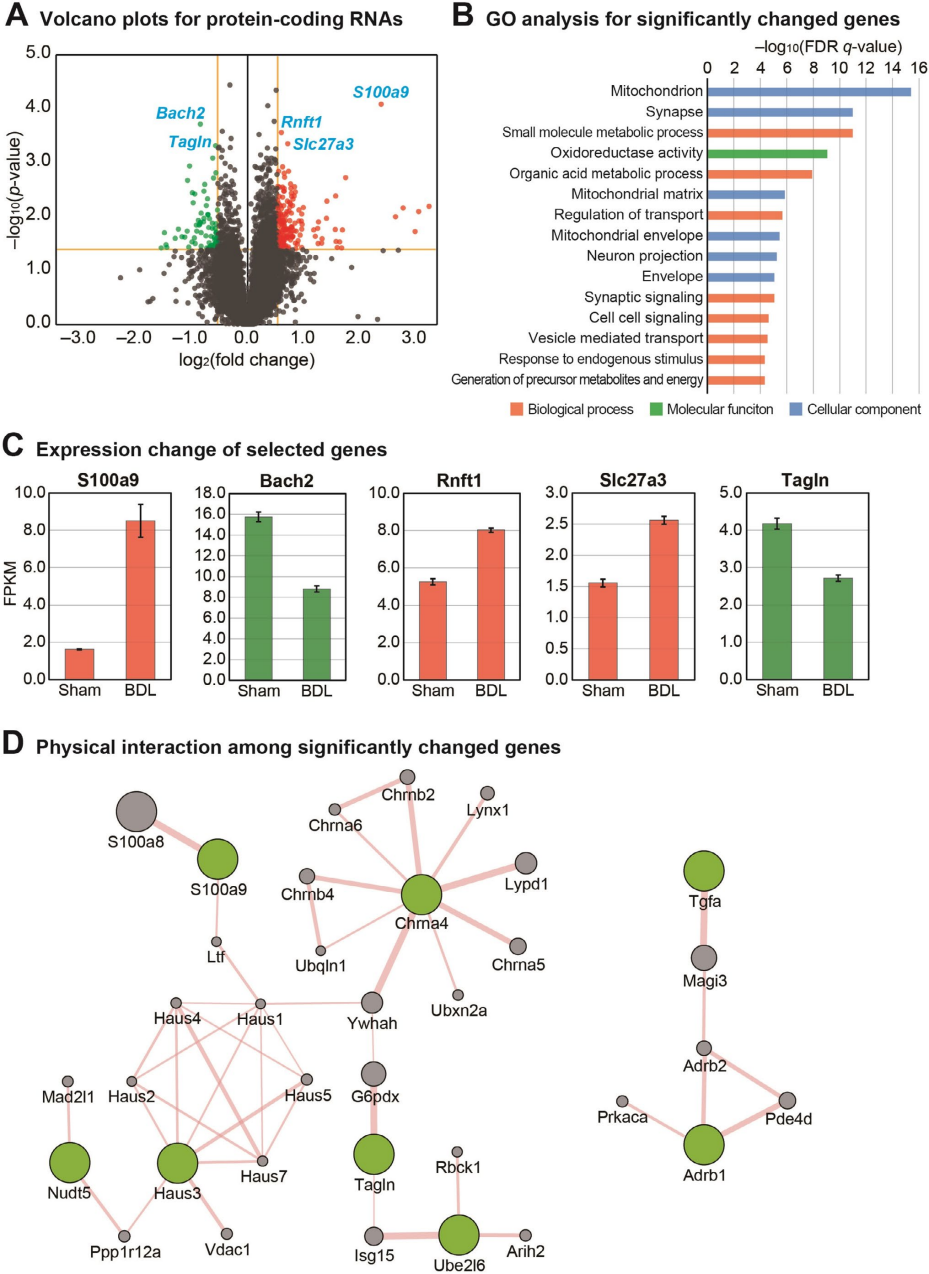
Transcriptomic analysis of differentially expressed protein‐coding RNAs in the olfactory bulb of bile duct ligation (BDL) mice. (A) Volcano plot depicting the protein‐coding RNA expression profile. Significantly altered genes (*p* < 0.05, absolute fold change > 1.5) are represented outside the yellow threshold lines. Red and green dots indicate significantly upregulated (159 genes) and downregulated (70 genes) transcripts, respectively. Genes with *p* < 0.001 are individually labeled. (B) Gene Ontology (GO) enrichment analysis of significantly altered protein‐coding genes (*p* < 0.01). The analysis was performed using the Molecular Signatures Database [29]. GO term categories are color‐coded: Biological process (red), molecular function (green), and cellular component (blue). (C) Expression profiles of the most significantly altered genes (*p* < 0.001 from panel A) comparing olfactory bulbs from sham and bile duct ligation (BDL) mice. (D) Physical interaction network analysis of differentially expressed protein‐coding genes. The 30 most significantly altered genes (based on *p* value) were analyzed using the GeneMANIA plugin in Cytoscape [33]. Only interaction clusters containing at least two input proteins are displayed. Input proteins are shown in green, whereas additional interacting proteins are shown in gray.

After characterizing the cellular effects of ammonia toxicity on olfactory bulb cells, we proceeded to profile transcriptome alterations in the olfactory bulbs of BDL mice, our established HE model. We compared gene expression patterns in olfactory bulbs from BDL mice with those from sham‐operated controls. Our analysis identified 229 significantly altered genes, comprising 70 downregulated and 159 upregulated genes (Figure 2A). Among the most significantly altered protein‐coding genes in the olfactory bulbs of BDL mice were BTB domain and CNC homolog 2 (*Bach2*), Transgelin (*Tagln*), Ring finger protein transmembrane 1 (*Rnft1*), S100 calcium‐binding protein A9 (*S100a9*), and solute carrier family 27 member 3 (*Slc27a3*) (Figure 2A). To understand the functional implications of these transcriptional changes, we performed GO analysis on the significantly altered genes. The GO analysis revealed enrichment of terms including mitochondrion, synapse, small molecule metabolic process, oxidoreductase activity, mitochondrial matrix, neuron projection, and synaptic signaling (Figure 2B). These enriched functional categories align closely with our cellular analysis findings (Figure 1), suggesting that olfactory bulbs in HE patients likely experience dysregulation of mitochondrial function, oxidative stress response, and synaptic plasticity. We focused particularly on the five most significantly altered genes—*S100a9*, *Bach2*, *Rnft1*, *Slc27a3*, and *Tagln*—all exhibiting expression changes with *p* values below 0.001 following BDL operation (Figure 2C). Based on these expression profiles and known gene functions, we hypothesize that olfactory bulbs in HE patients likely experience increased oxidative stress, altered inflammatory and immune responses, and compromised synaptic plasticity. This hypothesis is supported by the reduced expression of *Bach2*, which regulates antioxidative responses [34], and increased expression of *S100a9*, which mediates inflammatory processes [35] (see Discussion for detailed functional implications). To explore potential functional interactions among the differentially expressed genes, we analyzed physical interaction networks for the 30 most significantly altered protein‐coding genes based on *p*‐values using the GeneMANIA plugin in Cytoscape. We focused on interaction clusters containing at least two input proteins (Figure 2D). Notably, we identified a substantial interaction cluster encompassing 6 of the 30 significantly altered genes, suggesting that the cellular pathway involving these genes may play a critical role during HE progression. One component of this cluster, HAUS augmin‐like complex subunit 3 (*HAUS3*), participates in neuronal microtubule organization [36], suggesting this pathway could significantly impact olfactory bulb function in BDL mice (elaborated in Discussion).

### 3.3 BDL Operation Changes the Expression of Long Noncoding RNAs (lncRNAs) and Circular RNAs (circRNAs) in the Olfactory Bulb Tissue

**FIGURE 3:**
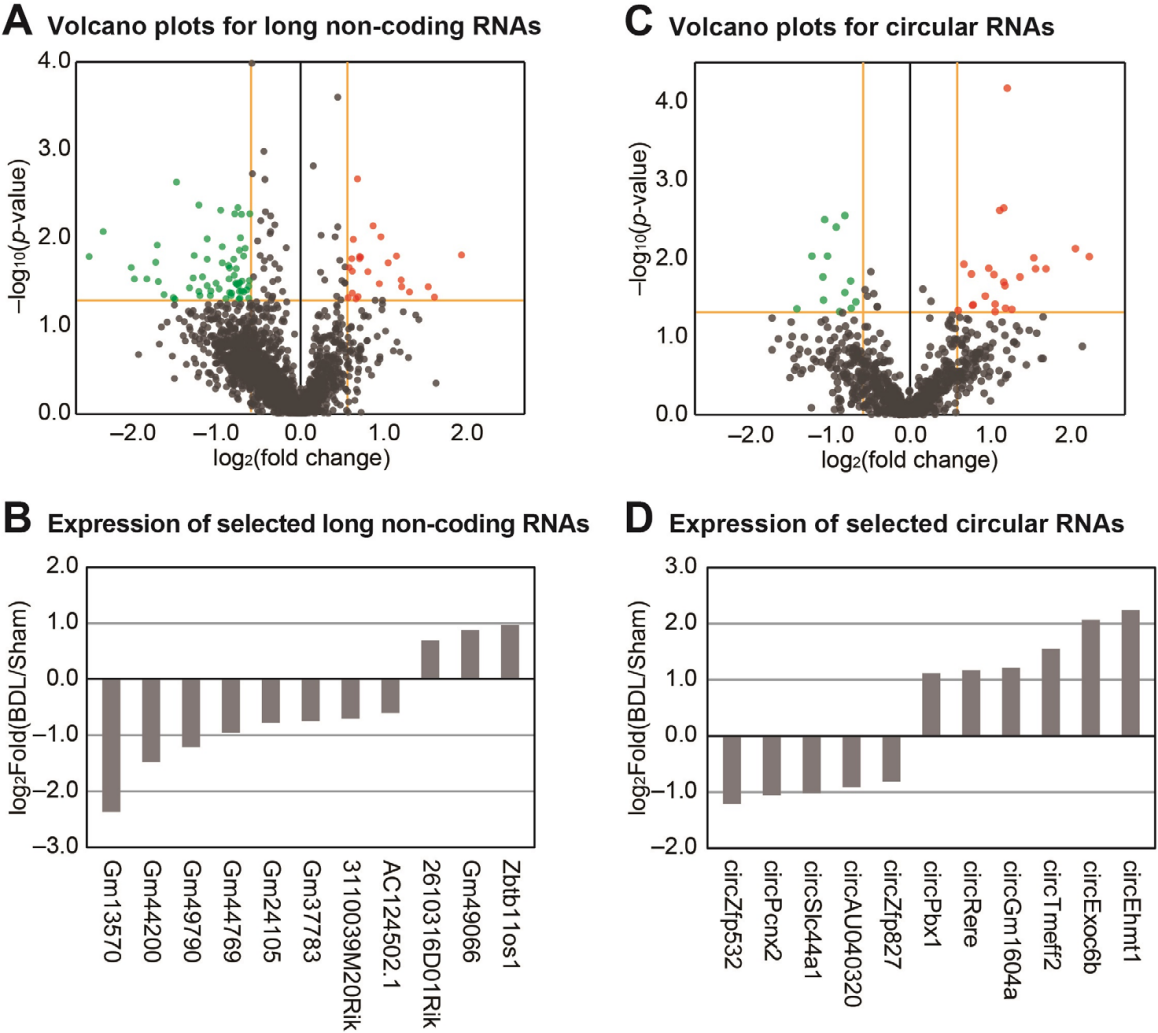
Differential expression of non‐coding RNAs in the olfactory bulb of bile duct ligation (BDL) mice. (A) Volcano plot showing long noncoding RNA (lncRNA) expression profiles. Significantly altered transcripts (*p* < 0.05, absolute fold change > 1.5) are represented outside the yellow threshold lines. Red and green dots indicate significantly upregulated (23 genes) and downregulated (59 genes) lncRNAs, respectively. (B) Fold changes of significantly altered lncRNAs (*p* < 0.01, absolute fold change > 1.5) between olfactory bulbs of sham and bile duct ligation (BDL) mice. We calculated fold change values based on the mean expression levels of three biological replicates in each group (*n* = 3 per group). (C) Volcano plot representing circular RNA (circRNA) expression profiles. Significantly altered transcripts (*p* < 0.05, absolute fold change > 1.5) are represented outside the yellow threshold lines. Red and green dots indicate significantly upregulated (23 genes) and downregulated (13 genes) circRNAs, respectively. (D) Fold changes of significantly altered circRNAs (*p* < 0.01) between olfactory bulbs of sham and BDL mice. We calculated fold change values based on the mean expression levels of three biological replicates in each group (*n* = 3 per group).

To comprehensively characterize the transcriptional landscape changes in olfactory bulbs during HE, we extended our investigation beyond protein‐coding RNAs to analyze differentially expressed noncoding RNA species in the BDL mouse model. Our transcriptome analysis identified substantial alterations in both lncRNAs and circRNAs. For lncRNAs, we detected 82 significantly altered transcripts, comprising 23 upregulated and 59 downregulated lncRNAs in the olfactory bulbs of BDL mice compared to sham controls (Figure 3A). To focus on the most biologically relevant changes, we selected lncRNAs with expression changes that met stringent criteria: *p* values below 0.01 and absolute fold changes exceeding 1.5 between BDL and sham mice olfactory bulbs (Figure 3B). These significantly altered lncRNAs represent promising candidates for further investigation into their regulatory roles during HE pathogenesis. Similarly, our analysis revealed substantial changes in circular RNA (circRNA) expression, with 36 significantly altered circRNAs identified in BDL mice olfactory bulbs compared to controls. This set included 23 upregulated and 13 downregulated circRNAs (Figure 3C). To prioritize biologically significant changes, we selected 11 circRNAs that exhibited both statistical significance (*p* < 0.01) and robust expression (average expression levels exceeding 5 FPKM across all samples), ensuring focus on abundant and reliably detected transcripts (Figure 3D). This comprehensive catalog of differentially expressed noncoding RNAs—both lncRNAs and circRNAs—provides valuable insights into the complex regulatory landscape changes in the olfactory bulb during HE pathogenesis. These identified ncRNAs constitute a valuable resource for future investigations into the molecular mechanisms underlying olfactory dysfunction in HE and may reveal potential therapeutic targets for intervention.

### 3.4 The Comparison of Gene Expression Profiles Between the Olfactory Bulb and Cerebral Cortex of BDL Mice

**FIGURE 4:**
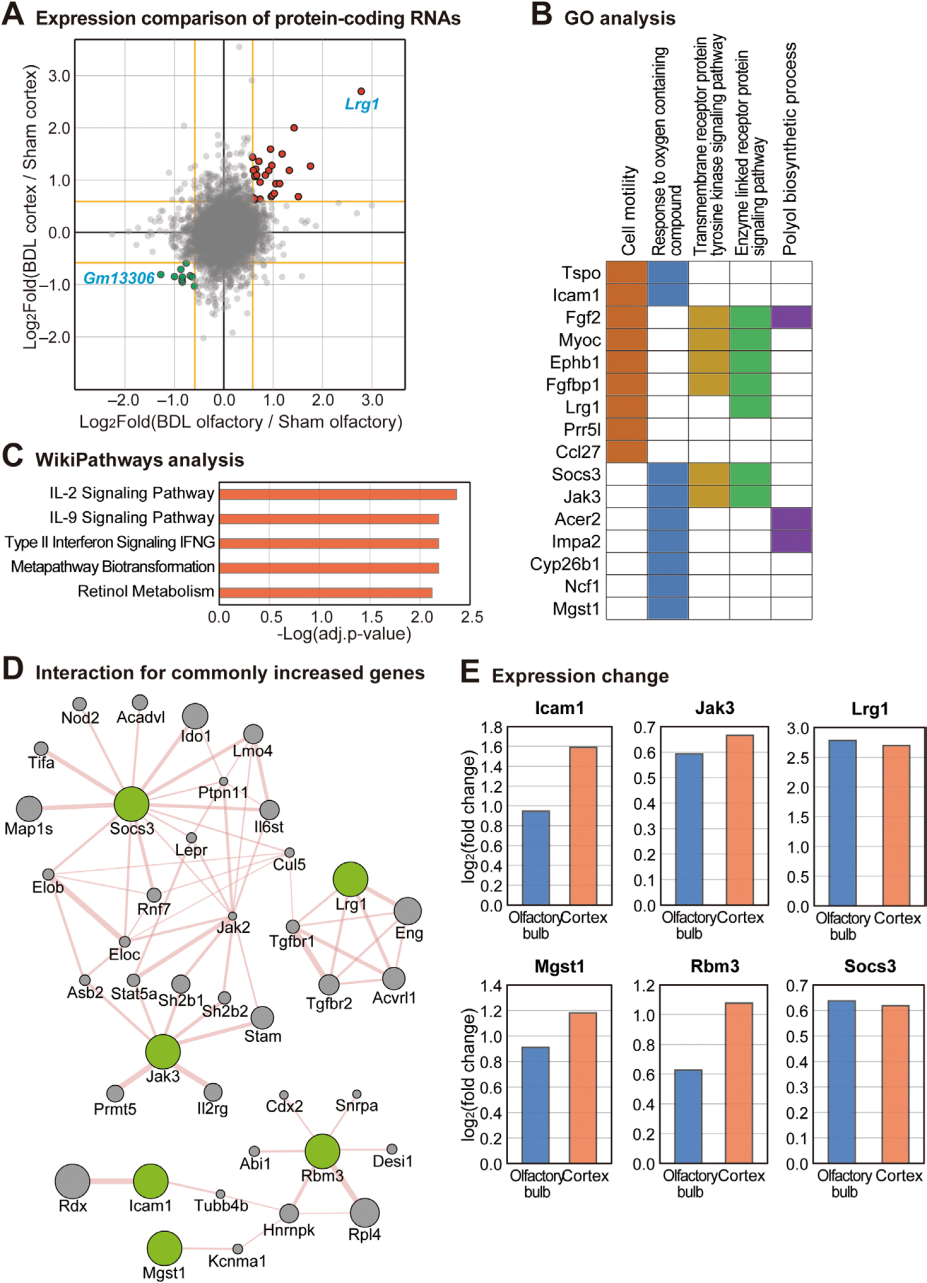
Comparative analysis of gene expression profiles between the olfactory bulb and cerebral cortex of bile duct ligation (BDL) mice. (A) Scatter plot comparing differential gene expression in olfactory bulb (x‐axis) versus cerebral cortex (y‐axis) from bile duct ligation (BDL) mice relative to sham controls. Genes with absolute fold change > 1.5 in both tissues are represented outside the yellow threshold lines. Red and green dots indicate consistently upregulated (27 genes) and downregulated (10 genes) transcripts across both brain regions, respectively. Leucine‐rich alpha‐2 glycoprotein 1 (*Lrg1*), the most highly altered gene in both data sets, is highlighted in blue. (B) Gene Ontology (GO) analysis of the 37 consistently altered protein‐coding genes identified in panel A, performed using the Molecular Signatures Database [29]. The five most significantly enriched biological process GO terms are shown with their constituent genes. (C) WikiPathways enrichment analysis of commonly increased genes. Bars represent the statistical significance of enriched pathways (−log_10_(Benjamini‐Hochberg adjusted *p* values)), based on the WikiPathways 2024 Mouse database. (D) Physical interaction network of consistently upregulated protein‐coding genes across both brain regions, visualized using the GeneMANIA plugin in Cytoscape [33]. Only interaction clusters containing at least two input proteins are displayed. Input proteins are shown in green, whereas additional interacting proteins are shown in gray. (E) Expression changes of six consistently upregulated input genes in olfactory bulb and cerebral cortex following BDL operation. The fold change values based on the mean expression levels of three biological replicates in each group were calculated and shown (*n* = 3 per group).

In our previous study, we characterized gene expression alterations in the cerebral cortex of BDL mice [19]. That investigation identified key roles for lncRNAs, including ZNFX1 Antisense RNA 1 (*ZFAS1*) and Growth Arrest Specific 5 (*GAS5*), in regulating apoptosis and neuronal structure in both the cerebral cortex of BDL mice and neuronal cells under hyperammonemic conditions [19]. To identify genes that might play conserved roles across different brain regions during HE, we performed a comparative analysis between the current olfactory bulb transcriptome data and our previously published cerebral cortex dataset (Figure 4). This cross‐regional comparison revealed 37 protein‐coding genes consistently dysregulated in both brain regions, comprising 27 commonly upregulated and 10 commonly downregulated genes. The most significantly upregulated gene across both regions was leucine‐rich alpha‐2 glycoprotein 1 (*Lrg1*), while *Gm13306* showed the most pronounced downregulation (Figure 4A). To understand the functional implications of these conserved transcriptional changes, we performed GO analysis on these 37 commonly altered genes (Figure 4B). The analysis identified five top enriched biological process categories based on *p*‐value: cell motility, response to oxygen‐containing compounds, transmembrane receptor protein tyrosine kinase signaling pathway, enzyme‐linked receptor protein signaling pathway, and polyol biosynthetic process (Figure 4B). To gain further insight into the functional relevance of the 27 commonly increased genes, we performed pathway enrichment analysis using the WikiPathways 2024 Mouse database via Enrichr [30, 31]. Notably, several immune‐related pathways were significantly enriched, including the IL‐2 signaling pathway, IL‐9 signaling pathway, and Type II interferon (IFNG) signaling (Figure 4C). These findings suggest that immune activation may be a shared transcriptomic feature across brain regions in the HE model. To further explore potential functional relationships among these conserved genes, we analyzed physical interaction networks for the significantly upregulated protein‐coding genes identified in both brain regions. We focused on interaction clusters containing at least two input proteins and identified two substantial interaction networks encompassing six genes from our dataset (Figure 4C). These six commonly upregulated genes were suppressor of cytokine signaling 3 (*Socs3*), *Lrg1*, Janus kinase 3 (*Jak3*), RNA binding motif protein 3 (*Rbm3*), intercellular adhesion molecule 1 (*Icam1*), and microsomal glutathione S‐transferase 1 (*Mgst1*) (Figure 4D). These consistently dysregulated genes likely play conserved roles in both the olfactory bulb and cerebral cortex during HE pathogenesis. Notably, *Lrg1* has been implicated in apoptosis regulation and cell differentiation, supporting our hypothesis regarding shared molecular mechanisms underlying HE‐induced neuronal dysfunction across brain regions [37, 38].

### 3.5 The Comparison of Gene Expression Profiles in Olfactory Bulbs Between BDL Mice and High‐Fat Diet (HFD) Mice

**FIGURE 5:**
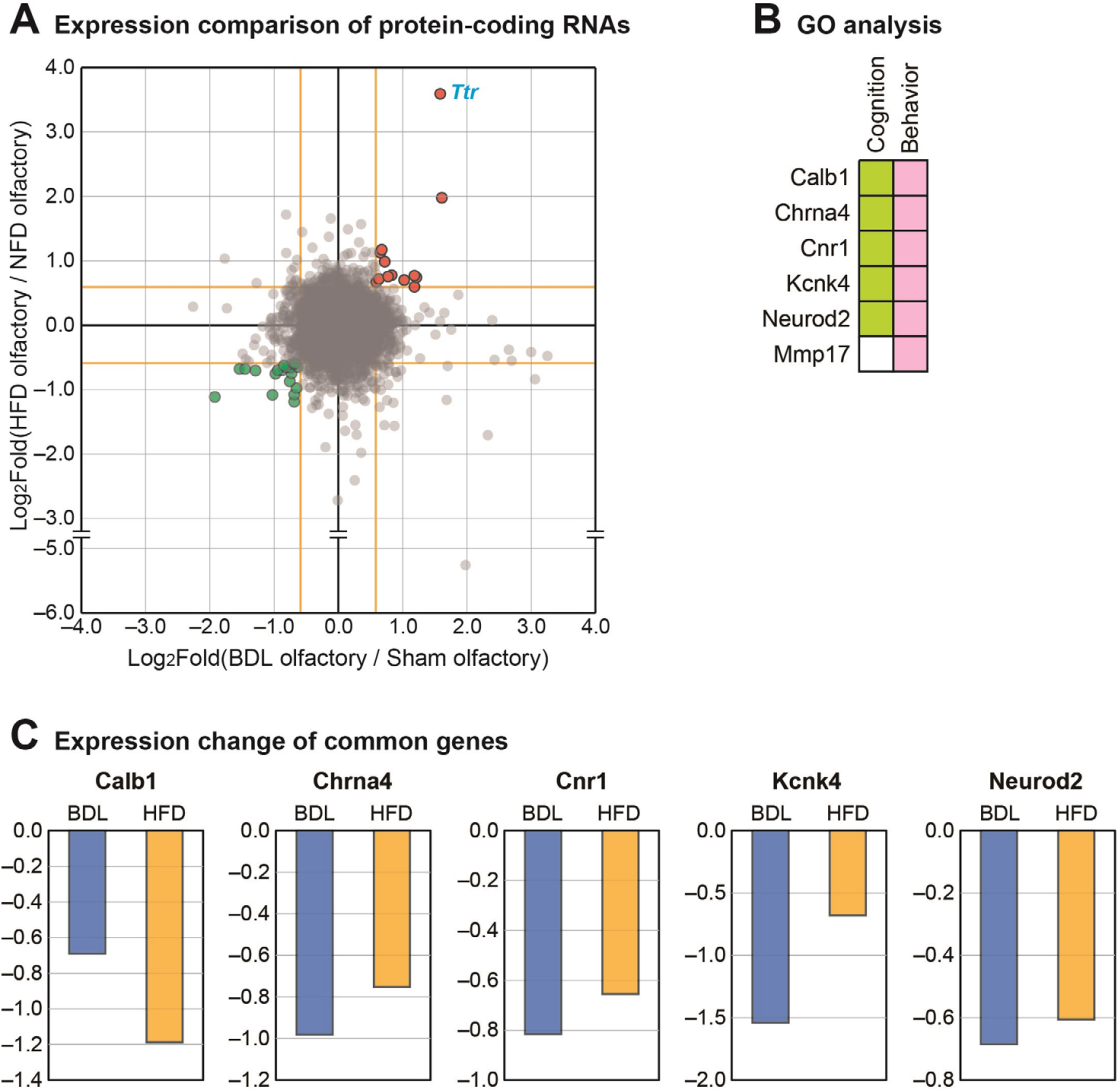
Comparative analysis of gene expression profiles in the olfactory bulbs between bile duct ligation (BDL) and high‐fat diet (HFD) mouse models. (A) Comparison of protein‐coding RNA expression changes in olfactory bulbs between bile duct ligation (BDL)‐operated and high fat diet (HFD)‐fed mice. Genes with absolute fold change > 1.5 in both models are represented outside the yellow threshold lines. Red and green dots indicate consistently upregulated (13 genes) and downregulated (20 genes) transcripts across both models, respectively. Transthyretin (*Ttr*), the most highly altered gene in both datasets, is highlighted in blue. (B) Gene Ontology (GO) analysis of the 33 consistently altered protein‐coding genes identified in panel A, performed using the Molecular Signatures Database [29]. The two significantly enriched GO terms are shown with their constituent genes. (C) Expression changes of the five protein‐coding genes common to both enriched GO terms in panel B, comparing their regulation in BDL‐operated and HFD‐fed mice. The fold change values based on the mean expression levels of three biological replicates in each group were calculated and shown (*n* = 3 per group).

In a previous investigation, we characterized the gene expression profile of olfactory bulbs in mice fed a high‐fat diet (HFD) compared to normal‐fed controls [39]. This earlier study demonstrated that metabolic imbalance induced by HFD consumption triggers substantial transcriptomic alterations in the olfactory bulbs. Specifically, we identified expression changes in genes related to olfactory function, including those governing neurogenesis, synapse formation, and neuron projection in HFD mice. Additionally, we cataloged various protein‐coding genes, lncRNAs, and circRNAs with altered expression in the olfactory bulbs of HFD‐fed mice [39]. Given that metabolic dysregulation is a common feature of both HFD‐induced obesity and BDL‐induced HE, we performed a comparative analysis of protein‐coding RNA expression changes in the olfactory bulbs across these two metabolic disorder models (Figure 5). This cross‐model comparison identified 33 genes with consistent expression changes, comprising 13 commonly upregulated and 20 commonly downregulated genes in both experimental paradigms. The most significantly altered gene across both models was transthyretin (*Ttr*), which has documented roles in axon outgrowth, synaptic vesicle dynamics, and cytoskeletal rearrangement [40]. This finding suggests that *Ttr* may serve as a key molecular player in olfactory dysfunction across diverse metabolic disorders (Figure 5A). To understand the functional implications of these shared transcriptional alterations, we performed GO analysis on the 33 consistently dysregulated genes. The analysis revealed two significantly enriched GO terms: cognition and behavior (Figure 5B). These enriched functional categories align with the observed sensory and cognitive impairments characteristic of both HE and HFD‐induced metabolic disorders. We further examined the expression patterns of five genes commonly represented in both enriched GO terms: calbindin 1 (*Calb1*), cholinergic receptor nicotinic alpha 4 subunit (*Chrna4*), cannabinoid receptor 1 (*Cnr1*), potassium two pore domain channel subfamily K member 4 (*Kcnk4*), and neuronal differentiation 2 (*Neurod2*) (Figure 5C). These genes exhibited consistent directional changes across both models, suggesting they may constitute a conserved molecular signature underlying olfactory dysfunction in metabolic disorders of diverse etiologies. The identification of these commonly altered genes across different metabolic disorder models suggests shared molecular mechanisms may drive olfactory dysfunction in both conditions, despite their distinct pathophysiological origins. These findings highlight potential therapeutic targets that could address olfactory impairments across multiple metabolic disease contexts.

## 4 Discussion

In this study, we investigated transcriptome alterations in the olfactory bulbs of BDL mice as a model of HE. Before performing transcriptome analysis, we first characterized the cellular responses of olfactory bulb cells to hyperammonemic conditions that mimic HE pathophysiology. Our in vitro experiments with OBC1 cells revealed multiple cellular pathologies: impaired mitochondrial membrane potential, enhanced cell death, excessive ROS production, and increased secretion of pro‐inflammatory cytokines under hyperammonemic conditions. Previous research has established that mitochondrial dysfunction in the olfactory bulb significantly impacts odor detection capabilities [41]. Additionally, ammonia has been shown to induce excessive ROS generation [42], which subsequently causes mitochondrial dysfunction and compromises neuronal activity [43]. Impaired mitochondrial membrane potential disrupts ATP production and electron transport chain functionality [44], creating an energy deficit critical for neuronal function. Autophagy constitutes a vital cellular mechanism for eliminating dysfunctional mitochondria and maintaining cellular homeostasis [45]. However, enhanced autophagy can also accelerate cell death under severe stress conditions [46]. Our observation of increased LC3B type II protein expression suggests that ammonia exposure triggers autophagy‐related cell death pathways in OBC1 olfactory bulb cells. This interpretation is further supported by our detection of elevated cleaved caspase‐3 and Bax levels, indicating activation of apoptotic pathways. These findings collectively suggest that in hyperammonemic environments characteristic of HE, olfactory bulb cells likely sustain significant damage, exhibiting elevated ROS production and mitochondrial dysfunction. Furthermore, our data indicate that the olfactory bulb in HE patients may experience enhanced production of pro‐inflammatory cytokines, including: (1) M‐CSF, which promotes neuroinflammation and microglial activation [47]; (2) MCP1, which enhances macrophage activation and immune cell recruitment [48]; and (3) TARC, which stimulates autoimmune responses and T‐cell activity [49]. Building on these cellular analyses, we examined gene expression alterations in the olfactory bulb tissues of BDL mice compared to sham‐operated controls. GO analysis of the differentially expressed genes highlighted changes in pathways governing mitochondrial function, oxidative stress responses, and synaptic plasticity. These findings align with previous research demonstrating that hyperammonemia induces mitochondrial dysfunction, neuronal cell death, and chronic oxidative stress throughout the central nervous system in HE patients [50, 51, 52]. Mitochondrial dysfunction resulting from BDL operation has been linked to impaired cerebral energy metabolism, decreased creatine kinase activity, compromised glucose uptake, and disturbed ATP/ADP ratios in HE [53]. Considering this evidence alongside our findings, we propose that cells in the olfactory bulbs of HE patients likely experience altered mitochondrial function, heightened oxidative stress, and impaired energy metabolism, ultimately leading to synaptic dysfunction and neuronal death that contribute to olfactory impairments.

Our transcriptome analysis revealed several significantly altered genes in the olfactory bulbs of BDL mice, including upregulation of S100 calcium‐binding protein A9 (*S100a9*), *Rnft1*, and *Slc27a3* and downregulation of *Bach2* and *Tagln*. *S100a9*, a member of the S100 proteins superfamily and a calcium‐binding protein also known as myeloid‐related protein 14 (MRP‐14) [54], exhibits elevated expression under various stress conditions in cell types including tumor cells, endothelial cells, microglia, and neurons [54]. Extracellular *S100a9* activates pro‐inflammatory responses through toll‐like receptor 4 (TLR4) and receptors for advanced glycation end‐products (RAGE) [55, 56]. Multiple studies have demonstrated that *S100a9* exacerbates neuroinflammation and regulates cellular migration, proliferation, and differentiation [38, 57]. In respiratory pathologies, *S100a9* promotes tissue remodeling of nasal epithelium in chronic rhinosinusitis with nasal polyps [58] and modulates neuroinflammation through TLR4‐dependent pathways [35]. Furthermore, increased *S100a9* expression enhances matrix metalloproteinase production associated with cell proliferation in chronic rhinosinusitis [59]. Based on the observed upregulation of S100a9 and its reported roles in other systems, we speculate that this gene may be involved in neuroinflammation and altered cell proliferation in the olfactory bulb. However, functional validation will be needed to establish its precise role in HE. Conversely, *Bach2* regulates neuronal differentiation in response to oxidative stress [60, 61], modulates CD8+ T‐cell differentiation by controlling AP‐1, and promotes expression of antioxidative stress genes [34, 61]. Recent research indicates that *Bach2* regulates Th2 immune response activity, influencing susceptibility to allergic rhinitis [62]. The decreased *Bach2* expression observed in our model suggests that HE patients may exhibit attenuated antioxidative responses and altered immune functions in the olfactory bulb. Our protein interaction network analysis revealed strong interactions between *S100a9* and S100 calcium‐binding protein A8 (*S100A8*). These Ca^2+^‐binding proteins form the S100A8/A9 complex involved in cytoskeletal rearrangement, microtubule network stabilization [63], inflammatory response modulation [64, 65], and promotion of pro‐inflammatory cytokine secretion [66]. Additionally, we identified numerous interactions involving the *HAUS3* protein. The HAUS complex, including *HAUS3*, plays critical roles in neuronal microtubule organization during axon outgrowth and regulates microtubule density in dendrites [36]. Other significant interaction proteins in our network include Nudix Hydrolase 5 (*Nudt5*), which maintains basal ATP levels and participates in DNA repair systems [67], and Cholinergic Receptor Nicotinic Alpha 4 Subunit (*Chrna4*), which modulates neural activity and is implicated in frontal lobe epilepsy development [68, 69]. These interaction data suggest that HE likely affects neurite outgrowth, neuronal network stabilization, inflammatory responses, mitochondrial ATP generation, and DNA repair mechanisms in the olfactory bulb. Our transcriptome analysis also identified significant alterations in the lncRNA *2610316D01Rik* (Figure 3). Interestingly, this lncRNA's promoter region overlaps with the promoter of protocadherin 10, a member of the cadherin superfamily [70]. Protocadherins are predominantly expressed in the central nervous system and localize to synapses [71]. Notably, protocadherin 10 deficiency leads to impaired axon projection and migration [70, 72]. As many lncRNAs sharing promoter sites with adjacent protein‐coding genes can regulate their expression, we hypothesize that *2610316D01Rik* may influence protocadherin 10 function and consequently affect neuronal connectivity in HE. In our comparative analysis of genes commonly altered in both the olfactory bulb and cerebral cortex of BDL mice, we identified *Lrg1* as a prominently upregulated transcript (Figure 4). *Lrg1* promotes obesity‐induced hepatosteatosis by enhancing de novo lipogenesis while attenuating fatty acid β‐oxidation and inhibits hepatic insulin signaling through interactions with insulin receptor substrates 1 and 2 [73]. This protein functions as a critical mediator in signaling interactions between adipocytes and hepatocytes under metabolic imbalance conditions [73, 74]. Within the central nervous system, *Lrg1* promotes cellular apoptosis and autophagy through the TGF‐β‐smad1/5 signaling pathway in response to oxidative stress‐induced brain injury [75]. Notably, overexpression of *Lrg1* in the hippocampus results in reduced synaptic vesicle density, contributing to cognitive impairment [37]. Furthermore, *Lrg1* modulates neuroinflammatory processes, apoptotic pathways, and nerve regeneration [76]. Based on these established functions and our observation of its consistent upregulation across brain regions, we speculate that elevated *Lrg1* expression may contribute to these processes in the context of HE, although further experimental validation is warranted. Our cross‐model comparison between BDL‐operated and HFD‐fed mice identified *Ttr* as the most significantly altered gene in the olfactory bulbs of both models (Figure 5). *Ttr* is primarily synthesized in the liver with additional production occurring in the choroid plexus of the brain [77]. This protein plays an essential role in transporting thyroxine from the liver to the cerebrospinal fluid (CSF) [77] and contributes significantly to cognitive function, nerve regeneration, and axonal outgrowth [40]. Previous research has demonstrated that mutations in the *Ttr* gene can lead to excessive protein aggregation into amyloid fibrils, ultimately resulting in transthyretin amyloidosis—a condition causing irreversible damage to multiple organs, including the brain [78]. Given our findings and the established functions of *Ttr*, we hypothesize that both HE patients and individuals with obesity‐related metabolic disorders likely experience alterations in *TTR* expression within the olfactory bulb, potentially affecting cognitive processing, nerve regeneration capacity, and axonal outgrowth. Further studies are needed to identify more specific mechanisms of noncoding RNAs in the olfactory bulb and to develop candidate RNA therapeutics and apply them clinically in HE patients.

While our study provides a comprehensive transcriptomic overview of olfactory bulb changes in a HE mouse model, we acknowledge several limitations. First, the functional roles of the differentially expressed ncRNAs and coding genes were inferred from prior literature or known molecular functions but were not experimentally validated in this study. Second, behavioral assays and in vivo functional studies were not conducted. Future work is needed to experimentally validate the roles of key ncRNAs and protein‐coding genes in HE‐related olfactory and cognitive dysfunction.

## Author Contributions

**Young‐Kook Kim:** conceptualization, data curation, formal analysis, investigation, methodology, validation, visualization, writing – original draft. **Sujung Yeom:** conceptualization, data curation, formal analysis, investigation, methodology, validation, writing – original draft. **Seo Yoon Choi:** data curation, formal analysis, investigation, methodology, validation, visualization. **Yeongseo Ryu:** formal analysis, investigation, methodology. **Dahee Jeong:** formal analysis, methodology. **Danbi Jo:** methodology. **Dong Hoon Lee:** conceptualization, data curation, formal analysis, funding acquisition, investigation, methodology, project administration, resources, validation, visualization, writing – original draft, Writing – review and editing. **Juhyun Song:** conceptualization, data curation, formal analysis, funding acquisition, investigation, methodology, project administration, resources, supervision, validation, visualization, writing – original draft, writing – review and editing. All authors read and approved the final manuscript.

## Conflicts of Interest

The authors declare no conflicts of interest.

## Supporting information


**Figure S1:** cns70596‐sup‐0001‐FigureS1.pdf.

## Data Availability

The data that support the findings of this study are available from the corresponding author upon reasonable request.
