# Supplementary figures and images for: The Change of Noncoding RNA Expression in Olfactory Bulb of Hepatic Encephalopathy Mouse Model: Transcriptomic Analysis and Cellular Analysis
Source: CNS Neurosci Ther. 2025 Sep 4;31(9):e70596. doi: 10.1111/cns.70596 (PMC12409072; doi:10.1111/cns.70596)

### Full uncropped blots

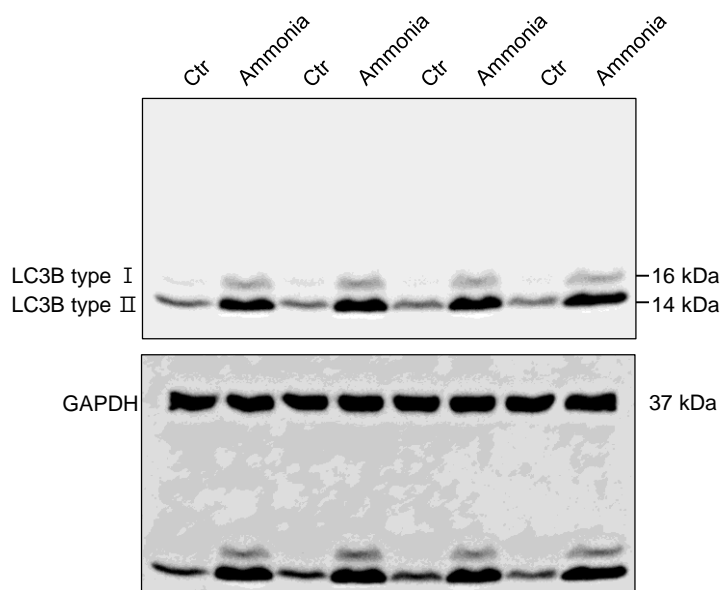

Supplement: Supplementary file 1 — Figure S1: cns70596‐sup‐0001‐FigureS1.pdf. [file CNS-31-e70596-s001.pdf]
